# MiR‐497‐5p inhibits cell proliferation and metastasis in hepatocellular carcinoma by targeting insulin‐like growth factor 1

**DOI:** 10.1002/mgg3.2114

**Published:** 2022-11-29

Xu, G. S., Li, Z. W., Huang, Z. P., Brundicardi, C. F., Jia, F., Song, C., Zou, H. J & Sun, R. F. “MiR‐497‐5p inhibits cell proliferation and metastasis in hepatocellular carcinoma by targeting insulin‐like growth factor 1”. *Molecular Genetics & Genomic Medicine*. 2019, 7(10), e00860. https://doi.org/10.1002/mgg3.860

The above article, published online on 23 August 2019 in Wiley Online Library (https://onlinelibrary.wiley.com), has been retracted by agreement between the authors, the journal Editor in Chief Dr. Suzanne Hart, and John Wiley & Sons. The retraction has been agreed due to problems in the original image preparation and data presentation, which undermine the reliability of the data presented as well as the article's conclusions.

